# Cardiovascular risk assessment in South and Middle-East Asians living in the Western countries

**DOI:** 10.12669/pjms.36.7.3292

**Published:** 2020

**Authors:** Sahrai Saeed, Alka M. Kanaya, Louise Bennet, Peter M Nilsson

**Affiliations:** 1 Sahrai Saeed, Department of Heart Disease, Haukeland University Hospital, Bergen, Norway; 2 Alka M. Kanaya, Department of Medicine and Epidemiology and Biostatistics, University of California, San Francisco, CA, USA; 3 Louise Bennet, Department of Clinical Sciences, Family Medicine, Lund University Malmo, Sweden; 4 Peter M Nilsson, Department of Clinical Sciences, Lund University, Skane University Hospital, Malmo, Sweden

**Keywords:** Cardiovascular risk, Caucasians, Diabetes, Hypertension, Metabolic Syndrome, Middle-East, South Asians

## Abstract

Nearly a quarter of the world population lives in the South Asian region (India, Pakistan, Bangladesh, Sri Lanka, Nepal, Bhutan, and the Maldives). Due to rapid demographic and epidemiological transition in these countries, the burden of non-communicable diseases is growing, which is a serious public health concern. Particularly, the prevalence of pre-diabetes, diabetes and atherosclerotic cardiovascular disease (CVD) is increasing. South Asians living in the West have also substantially higher risk of CVD and mortality compared with white Europeans and Americans. Further, as a result of global displacement over the past three decades, Middle-Eastern immigrants now represent the largest group of non-European immigrants in Northern Europe. This vulnerable population has been less studied. Hence, the aim of the present review was to address cardiovascular risk assessment in South Asians (primarily people from India, Pakistan and Bangladesh), and Middle-East Asians living in Western countries compared with whites (Caucasians) and present results from some major intervention studies. A systematic search was conducted in PubMed to identify major cardiovascular health studies of South Asian and Middle-Eastern populations living in the West, relevant for this review. Results indicated an increased risk of CVD. In conclusion, both South Asian and Middle-Eastern populations living in the West carry significantly higher risk of diabetes and CVD compared with native white Europeans. Lifestyle interventions have been shown to have beneficial effects in terms of reduction in the risk of diabetes by increasing insulin sensitivity, weight loss as well as better glycemic and lipid control.

## INTRODUCTION

The risk of cardiovascular disease (CVD) events differs according to ethnical background. Some countries in the East Asian region (Japan and China) have lower risk of CVD compared with South Asians and Europeans.^1^-^3^ On the other hand, people of African origin have a higher prevalence of hypertension and stroke compared with Europeans.^2^,^4^,^5^ South Asian populations have generally a higher burden of type 2 diabetes (T2D) and CVD than other race/ethnic groups, and the prevalence of both conditions is increasing.^6^-^8^ The cardiovascular (CV) risk profile of South Asians, particularly individuals from the Indian subcontinent (India, Pakistan and Bangladesh) living in the United Kingdom (UK), Canada and United States (US), has been investigated in a number of observational studies.^9^-^15^ These studies show that South Asians living in Western countries have a higher risk of coronary artery disease (CAD), insulin resistance, T2D, metabolic syndrome, hypertension, and dyslipidemia than whites.^8^-^15^

It is postulated that an increased genetic susceptibility to T2D, insulin resistance or impaired insulin secretion, unhealthy lifestyle, chronic inflammation and (abdominal-) obesity may contribute to the observed higher CVD risk in South Asian populations.^16^ Further, it has also been shown that compared to South Asians who remain in their native country, migrants from South Asia are characterized by higher BMI and CVD risk despite the fact that they share the same genetic background and cultural risk factors.^8^,^17^ It is also known that the Middle-Eastern populations have considerably higher prevalence of T2D in the cities than in the rural areas, probably due to easy access to high-calorie foods and sedentary lifestyles in city dwellers compared to those in the rural farming areas.^18^,^19^

In the present review, we have addressed CV risk assessment in South Asians, mainly people from India, Pakistan and Bangladesh living in Western countries, as well as Middle-East Asians living in Scandinavia, compared with white Europeans. In addition, the applicability of CV risk assessment tools, and proposed diagnostic criteria for metabolic syndrome, in various ethnic groups will be briefly summarized. Finally, the impact of lifestyle modification on CV health in these high-risk populations will be briefly addressed.

### Previous work on the risk assessment in South Asians compared with whites

Results from the Mediators of Atherosclerosis in South Asians Living in America (MASALA) study demonstrated that Indians in the US had higher adjusted odds of T2D than other ethnic groups despite having lower BMI and higher socioeconomic status.^12^ Compared to whites, the prevalence of hypertension (43 vs. 39%) and T2D (21 vs. 6%) was significantly higher in South Asian Indians after adjusting for several risk factors and confounders. In a previous MASALA pilot study analysis, the prevalence of metabolic syndrome was 41% in South Asians versus 30% in Whites.^13^ A disproportionately high prevalence of metabolic syndrome, lower β-cell function and higher insulin resistance in South Asians may explain the observed higher prevalence of T2D, endothelial dysfunction and pre-clinical and clinical atherosclerotic CVD.^8^ Of note, the higher prevalence of T2D in South Asians was not explained by traditional CV risk factors.^12^ Furthermore, the London Life Science Prospective (LOLIPOP) study by a research group in Harrow and London provided also important insights on the health status of Indian Asians in the UK.^20^,^21^ The LOLIPOP study compared 1058 European whites with 1353 Indian Asians and showed that the prevalence of CVD was 2-fold higher in Indian Asians than in European whites.^21^ Echocardiographic data of the LOLIPOP study also showed that Indian Asians had lower systolic and diastolic tissue Doppler velocities (subclinical LV dysfunction), higher filling pressure, and were more likely to have concentric remodeling (lower LV mass and LV volumes, but higher relative wall thickness).^20^ In another LOLIPOP sub-study, BMI did not differ between the groups but waist-hip ratio was significantly higher in South Indians compared with white Europeans 21 indicating body fat distribution differ across ethnicities. Although total cholesterol and LDL cholesterol were higher in European whites, Indian Asians had higher triglycerides and lower high-density lipoprotein (HDL) cholesterol, suggesting a distinct phenotype of atherogenic dyslipidemia, which is associated with an increased risk of CAD. This trend was also documented in other studies from Norway, Sweden, Germany and New Zeeland comparing the risk of CVD of immigrants from the Middle East and India with white Europeans.^22^-^25^ Immigrants from the Middle East had also lower blood pressure and total cholesterol, but higher triglycerides and lower plasma HDL levels.^22^-^25^

Furthermore, in the LOLIPOP study, based on adjusted statistical model, carotid intima-media thickness (IMT) and the prevalence and echogenicity of carotid plaque (soft vs. hard/calcified) did not differ between the two ethnic groups.^21^ The authors speculated that there might be other pathophysiological mechanisms for CVD in the high-risk Indian Asians.

T2D is a major driver of early and aggressive atherosclerosis in South Asians. Of note, CAD often appears as a complication of T2D, which is associated with adverse CV outcomes.^26^ In the MASALA study, South Asian men had higher coronary artery calcium (CAC) scores than African Americans, Latinos, and Chinese, but comparable with those of non-Hispanic whites while CAC did not differ among women in the five race/ethnic groups.^27^ In addition, distinct features of coronary artery size and disease severity (smaller cross-sectional area and more often involving multiple coronary vessels), have been revealed by conventional coronary angiography in South Asians compared to whites.^28^ CAC score is now increasingly used both in middle-aged high-risk patients and in younger patients with intermediate risk to assess coronary calcium score and the anatomy of coronary arteries.

The use of CAC in the CV risk assessment, and possibly CT angiography, may be particularly relevant for South Asian individuals who are younger and more often have T2D compared with their European and American counterparts, as well as a growing field of research.^8^

### South Asians and Middle Eastern immigrants in Scandinavia

South Asians in Norway, particularly immigrants from India and Pakistan, have been shown to be prone to T2D, abdominal obesity and other cardiometabolic disorders, which all substantially increase the risk of CVD events.^29^,^30^ However, it is important to highlight that not only people from South Asia, but also other non-Western populations such as immigrants from the Middle-East, run a higher risk of obesity, T2D, metabolic syndrome and CAD compared to native white North Europeans.^23^ Furthermore, in a large cohort study from Denmark comparing 62,461 non-western immigrants with 249,839 native Danes as a reference population (matched individually 1:4 for age and sex), multiple inter-ethnic trends in the risk of CVD were observed.^31^ Immigrants from Central Asia, South Asia, Iraq, Turkey, the Middle East and North Africa, Eastern Europe and the Former Yugoslavia all had significantly higher incidences of CAD compared with Danish-born people. Among people from East Asia and the Pacific, sex differences existed in the risk of CAD; women did not differ significantly from native Danes, while men had a significantly lower incidence of CAD compared with Danish-born people. Migrant status and income affected the risk of CAD (family-reunified immigrants had lower incidence of CAD than first-generation refugees).^31^ Similarly, data from the Cohort of Norway (CONOR) study of 62,145 participants aged 40-65 years showed that the risk of CVD was high in immigrants from South Asia (Indian subcontinent) and low in immigrants from East Asia.^2^,^3^ Finally, studies from Sweden have shown that long-term exposure to neighborhood deprivation also increased the risk of T2D in non-European refugees.^32^

### The applicability of CV risk assessment tools in various ethnic groups

A wide number of population-specific risk calculators have been established in the past, among others the Framingham Risk Score (FRS), the European SCORE (Systematic Coronary Risk Estimation), the AHA/ACC pooled cohort equation (PCE), and FINRISK (Finland Cardiovascular Risk Study).^8^,^33^ The SCORE system is widely used both in high- and low-risk European populations.^33^ Of note, these risk assessment tools are not derived from data originating from high-risk South Asians (who often are younger at time of disease onset than other ethnic groups) and therefore may underestimate their risk. Also, it is important to highlight that elevated waist-hip-ratio or waist circumference, which is common in South Asians, is not included in the SCORE or Framingham risk models, which may also underestimate the risk of CVD in South Asians. An adjusted methodological variant of the FRS by multiplying by 1.4 to 1.5 for South Asians has been proposed.^34^ Some earlier National Institute for Health and Care Excellence (NICE) guidelines in the UK also recommended an ethnicity-adjustment of FRS by multiplying in 1.4 for South Asians men.^35^ Another UK study also found that the FRS underestimated risk in South Asian women, but performed reasonably well in South Asian men when the score was multiplied by a factor of 1.4.^36^ The authors suggested that a risk multiplier for the FRS might also be applied for South Asian women. The same study also showed that there were inconsistencies in the performance of both Framingham and QRISK2 scores in terms of identifying high-risk individuals in South Asians and African-Caribbean populations.^36^ Although QRISK2 score included adjustment for socioeconomic deprivation and ethnicity, and was internally validated in the UK, its validation in other multi-ethnic datasets was strongly advised. Furthermore, the performance of the FRS 5-years CVD risk score was also tested in Indians living in New Zealand. The Framingham risk model performed relatively well in Indian men, but overestimated risk in Indian women (predicted risk values of about ≥6%) and of European New Zealanders of both sexes.^37^ A possible explanation for this apparent discrepancy with other studies was explained by the fact that medical treatment in high-risk South Asians in the study might have modified their risk of CVD. In addition, the New Zealand population was described as a low-risk population with declining risk of CAD and stroke. In the CONOR study from Norway, ethnic differences in overall CVD risk was lower when the NORRISK model was applied and greater with Framingham model.^2^ Finally, some studies from the US have also evaluated the ACC/AHA PCE in clinical populations using electronic health records in large health systems.^38^,^39^ In both studies, the PCE overestimated the risk of atherosclerotic CVD events in South Asians^38^ and many other ethnic populations.^39^ Hence, there are inconsistencies in the risk-predictive value of commonly used risk stratification models across various ethnic groups and none of them is universally applicable. An ideal CV risk model for South Asian population should be preferably derived and validated within South Asian populations.^40^

### The definition of Metabolic syndrome and abdominal obesity

In the harmonized criteria by the joint International Diabetes Federation/American Heart Association/National Heart, Lung, and Blood Institute/World Heart Federation and International Atherosclerosis Society criteria in 2009, an ethnicity-specific cut-off for waist circumference was included.^41^ According to these criteria, any three of the five criteria presented in Table-I are required for diagnosis of MetS:^41^

**Table-I:** Harmonized criteria for clinical diagnosis of the metabolic syndrome.^41^

Criteria		Definition
1	High waist circumference	Population-specific cutoff*
2	Elevated serum triglycerides Or drug treatment for hypertriglyceridemia	≥150 mg/dL (1.7 mmol/L)
3	Low HDL cholesterol	<40 mg/dL (1 mmol/L) in men
		<50 mg/dL (1.3 mmol/L) in women
4	Elevated office/clinic BP Or use of antihypertensive medications	Systolic BP ≥130 mmHg and/or diastolic BP ≥85 mmHg
5	Elevated fasting blood glucose Or taking anti-diabetic medications	≥100 mg/dL (5.6 mmol/l)

BP, blood pressure; HDL, high-density lipoprotein. *Asian (applicable for South Asians): ≥90 cm in men and ≥80 cm in women. *American and European-Caucasian: ≥102 cm in men and ≥88 cm in women.

The impact of Migration and Ethnicity on Diabetes in Malmo (MEDIM) study compared the risk of CVD in native Swedes with Middle East immigrants. The insulin sensitivity index in Swedes with central obesity (defined as waist circumference ≥94 cm in men and ≥80 cm in women accordance with the World Health Organization and International Diabetes Federation criteria) corresponded with waist circumference cut-offs of 84 cm in Iraqi men and 71 cm in women.^42^ The authors indicated that a roughly 10 cm lower cut-off values for abdominal obesity, than currently recommended by international guidelines, should be considered when estimating the risk of T2D in Middle Eastern populations.^42^ Other studies have also indicated that the risk of T2D in non-whites is equivalent to a lower waist circumference and BMI than proposed for whites. The BMI cut-off of 30 kg/m^2^ for the definition of obesity in whites corresponds to much lower cut-offs in other ethnic groups: 22 kg/m^2^ for South-Asians and 26 kg/m^2^ for black African populations 43. The MEDIM study group also showed that in individuals with normal BMI (<25 kg/m^2^), 21% of Iraqis and 9% of Swedes had insulin resistance.^42^ The corresponding figures were 28% in Iraqis and 9% in Swedes among individuals with normal waist circumference (men <94 cm, women <80 cm). The insulin sensitivity index for obese Swedes (BMI 30 kg/m^2^) corresponded with a BMI of 28.5 kg/m^2^ in Iraqi men and 27.5 kg/m^2^ in Iraqi women.^42^

Hence, in view of these differences in body size across various ethnic groups, as well as inconsistency in the risk prediction score, particularly in South Asians, larger collaborative studies are needed in the future to: 1) develop optimal risk stratification tools for these high-risk populations; 2) to explore the true prevalence and correlates of metabolic syndrome and; 3) study causality of metabolic syndrome with target organ damages and differences in incidence of cardiometabolic manifestations across ethnicities.

### Other Measures of Vascular health

The assessment of vascular disease, such as arterial stiffness, as estimated by aortic pulse wave velocities, is a less studied risk marker in South Asian populations compared to East Asians and whites.^44^ While individual components of CV risk factors such as elevated blood pressure and the circulating biomarkers, like plasma glucose and serum lipid levels, may fluctuate over time, and the recorded values at the time of risk assessment may not accurately reflect the true CV risk burden in individual patients. However, arterial stiffness is considered as a cumulative measure of previous exposure to CV risk factors, and represents an intermediate stage between CV risk factors and clinically overt CV event.^45^,^46^ Currently, the carotid-femoral pulse wave velocity measured by an applanation tonometry is the gold standard method for assessing arterial stiffness, and is an independent predictor of CVD and CV mortality, both in healthy populations and in high-risk populations.^45^,^46^ More investigation of arterial stiffness and distensibility are needed in South Asians and Middle Eastern populations to determine if these measures can add to atherosclerotic CVD risk estimation.

### Lifestyle intervention

In the large Prospective Urban Rural Epidemiology (PURE) study of 155722 participants from a wide number of countries according to the socio-economic status (low-income countries: Bangladesh, India, Pakistan, Tanzania, and Zimbabwe; middle-income countries: Argentina, Brazil, Chile, China, Colombia, Iran, Malaysia, Palestine, Philippines, Poland, Turkey, and South Africa; high-income countries: Canada, Saudi Arabia, Sweden, and United Arab Emirates) showed that approximately more than two-thirds of CVD and CV mortality were attributed to modifiable cardiometabolic risk factors (hypertension, abdominal obesity and elevated non-HDL cholesterol).^47^ Of note, the association between household air pollution, poor diet, low education, and low grip strength with CVD was stronger in middle-income and low-income countries compared with high-income countries. Hence, modification of CV risk factors by lifestyle interventions as well as therapeutic approaches are essential to avoid early CVD and mortality.

Lifestyle interventions with diet and physical exercise are effective measures in delaying and/or preventing the onset of T2D and improving overall health status in high-risk individuals.^48^ Studies from China 49, Finland 50, Sweden 51 and the US 52-53 have previously established the role of lifestyle modification (weight loss and active physical training) in T2D prevention. In the Finnish Diabetes Prevention Study of 522 overweight subjects with impaired glucose tolerance, lifestyle intervention by diet, weight loss and physical activity, reduced the risk of T2D by 58%.^50^

Overall, few culturally adapted lifestyle intervention studies have been conducted in people with minor ethnic background in Europe, and mainly the South Asian immigrants have been the target populations in these studies such as the PAMH (Physical Activity and Minority Health) study on Pakistani men^54^, and Innva-Diab Deplan study among Pakistani women in Norway,^55^ study on Surinamese immigrants in Netherlands^56^ and the Podosa trial in UK.^57^ Most of these studies^54^,^55^,^57^ suggested that culturally adapted education programmes improve cardiometabolic risk factors and reduces the risk of T2D.

The MEDIM population-based study conducted 2010 to 2012 in Malmo Sweden showed that Middle Eastern immigrants were physically less active and were at an increased risk for T2D.^58^,^59^ High levels of anxiety and depression were strongly associated with physical inactivity in this vulnerable population. In next step, individuals in the population based study identified at high risk of T2D (body mass index ≥28 kg/m^2^, and/or waist circumference ≥80 cm in women, and ≥94 cm in men, and/or pre-diabetes) were invited to participate in the MEDIM intervention study. Those accepting participation were randomized to either active intervention or control arm. Lifestyle intervention had significant favorable effects on cardio-metabolic risk factors and led to increased insulin sensitivity, reduction in body weight and low-density lipoprotein cholesterol in the intervention group compared to the control group.^58^ Those participants succeeding in changing lifestyles experienced positive support from their family relatives in their efforts.^60^ In addition, the study showed beneficial effects on mental health.^61^

Finally, the use of secondary prevention therapies and the impact of socioeconomic factors such as rural residence, education level and household wealth on CVD were studied in a PURE sub-study, confined only to South Asian population (India, Pakistan and Bangladesh).^62^ The authors concluded that in patients with CVD (CAD and stroke), the use of secondary preventive drugs was low; i.e. over 80% received none of the effective drug treatments, and low household wealth was the most important determinant.^62^ Hence, these results highlight the importance of optimal secondary preventive strategies alongside primary prevention with lifestyle modification.

### Perspectives

The development of targeted preventive strategies in high risk population i.e. South Asians and Middle-Eastern Asians, is of paramount importance. However, the optimal risk prediction, valid in all ethnic groups, is the cornerstone of these strategies. It is also important to highlight that the majority of studies exploring CV health in South Asians were based upon finding in first-generation migrants. These results should be carefully interpreted when applying to future generations.

South Asian labor migrants to the Middle East, especially in Gulf countries, have also a high burden of cardio-metabolic risk factors.^63^ This particular group comprised of young and vulnerable individuals deserves more attention in terms of a systematic CV screening, preventive strategies and a better/timely access to health services in order to avoid premature CV death.^63^ Finally, in the Central Asian countries including former Soviet republics, the burden of CVD is not well explored. A recent study showed that an upward trend, and high levels of premature avertable mortality from non-communicable diseases were reported by some World Health Organization (WHO) centers in the regions.^64^ Among these, Afghanistan, Uzbekistan and Turkmenistan represented Central Asia, Mongolia East, and Pakistan South Asia. Hence, a systematic CV screening for non-communicable diseases is highly needed in these countries in order to avoid premature CV deaths.

## CONCLUSIONS

People from South Asia and Middle East living in the West are characterized by a significantly higher CVD burden compared to white European populations. This may be due to disproportionate risk of T2D and other cardiometabolic risk factors, further exacerbated by poor lifestyle behaviors and socioeconomic factors. There are differences in the cut-offs of elevated waist-circumference, general obesity and metabolic syndrome across the ethnic groups. Inconsistencies exist in the validation of existing risk stratification tools across various ethnic groups. Ideally, a CV risk model should be derived and validated within South Asian and Middle Eastern populations. Further research into vascular health of high-risk populations should involve assessment of arterial stiffness and detection of subclinical target organ damage including left ventricular structure and function, and CAC score, if resources are available. Effective and timely lifestyle intervention with physical activity and diet can reduce the risk of T2D which may also avoid premature CV death.

### Authors Contribution

**SS and PMN** wrote the first draft of the article, which was subsequently revised and appraised on several occasions by AMK and LB.

All authors approved the final submission.

**SS** takes the responsibility and is accountable for all aspects of the work in ensuring that questions related to the accuracy or integrity of any part of the work are appropriately investigated and resolved.
